# Syndrome of Inappropriate Antidiuretic Hormone Secretion-Induced Encephalopathy in a Patient With COVID-19

**DOI:** 10.7759/cureus.16671

**Published:** 2021-07-27

**Authors:** Mohammed Elmahal, Petras Lohana, Priyanka Anvekar, Syed R Ali, Sherif A Baath Allah

**Affiliations:** 1 Internal Medicine, University of London, London, GBR; 2 Internal Medicine, Liaquat University of Medical and Health Sciences Hospital, Karachi, PAK; 3 Internal Medicine, Mahatma Gandhi Mission (MGM) Medical College and Hospital, Mumbai, IND; 4 Internal Medicine, Dow University of Health Sciences, Civil Hospital Karachi, Karachi, PAK; 5 Pediatrics, Masafi Hospital, Alexandria, EGY

**Keywords:** covid 19, acute hyponatremia, siadh, hyponatremic encephalopathy, interleukin (il)-6

## Abstract

Various electrolyte imbalances have been documented in coronavirus disease 2019 (COVID-19) patients who progress to severe acute respiratory syndrome coronavirus-2 infection. Patients with co-morbidities like diabetes, hypertension, obesity, ischemic heart disease, chronic kidney disease, and chronic obstructive pulmonary disease are more vulnerable to developing complications in the form of electrolyte disturbance.

We report a case of acute severe hyponatremia in a middle-aged man who was admitted to the hospital with viral pneumonia due to a coronavirus-2 infection. A dramatic drop of plasma sodium was preceded by gastrointestinal symptoms and followed by encephalopathy. On clinical assessment his plasma sodium was found to be critically low, i.e. 105 mmol/L. His chest x-ray showed minimal pleural effusion.

The patient was managed in the ICU and his serum sodium was normalized gradually with partial but rapid correction of this severe hyponatremia with hypertonic sodium chloride and followed by fluid restriction.

## Introduction

The first cases of a novel coronavirus, which was named later as coronavirus disease 2019 (COVID-19), were primarily detected in Wuhan, Hubei Province, China, in December 2019. It is caused by severe acute respiratory syndrome coronavirus 2 (SARS-CoV-2). The main routes of transmission are droplet and direct contact [1]. Presently, detection of the virus RNA using real-time polymerase chain reaction (RT-PCR) is the principal test applied to diagnose COVID-19 [2]. It is well known that COVID-19 can be associated with multiple electrolyte imbalances, particularly hyponatremia, hypokalemia, and hypocalcemia [3]. Significant hyponatremia is associated with severe outcomes, so rapid diagnosis using a systemic approach to narrow the differential diagnosis is vital. We present this case of syndrome of inappropriate antidiuretic hormone secretion (SIADH) due to viral pneumonia, in which hyponatremia developed in a relatively short time from a normal baseline level of serum sodium leading to deranged mental status in the patient. We intend to highlight the mechanism of SIADH-induced encephalopathy and its management.

## Case presentation

A 54-year-old man presented to the primary care with a sore throat, dry cough, body aches, and low-grade fever for three days. He was treated symptomatically; infection with SARS-CoV-2 was highly suspected. Hence a COVID-19 PCR swab was taken, and he was advised for home quarantine. On the next day, the patient was shifted to the hospital as he started to have shortness of breath and his result turned to be positive for COVID-19. The patient reported no chest pain, palpitation, or syncope. He had no complaints of headache, neck stiffness, dizziness, or muscle weakness. He also reported no abdominal pain, nausea, vomiting, diarrhea, or constipation. 

The patient’s medical history was notable for type 2 diabetes mellitus and hypertension; he is on sitagliptin 50 mg/metformin 1000 mg twice a day, amlodipine 5 mg/valsartan 160 mg tablet a day, and has no known drug allergies. He works as a salesman at a private company. No one in his family has a history of a similar condition. His mother and father had a history of diabetes and hypertension. He denied any recent travel to areas of high spread for COVID-19 or contact with people with similar symptoms. 

On examination, the patient was alert, orientated, and cooperative. He was able to speak in full sentences. He was slightly tachypneic, the respiratory rate 22/minute, the oxygen saturation was 97% while on 5 liters of oxygen through a simple face mask, the blood pressure 135/85 mmHg, the body temperature was 37.6 °C, and the heart rate was 92 beats per minute. His body mass index was 25.5 kg/m2.

Chest examination revealed good air intensity bilaterally and few bi-basal coarse crackles. The first and second heart sounds were normal, and no murmurs, gallop, or rubs. The abdomen was soft, not distended, with normal bowel sounds and no hepatosplenomegaly. 

**Figure 1:**
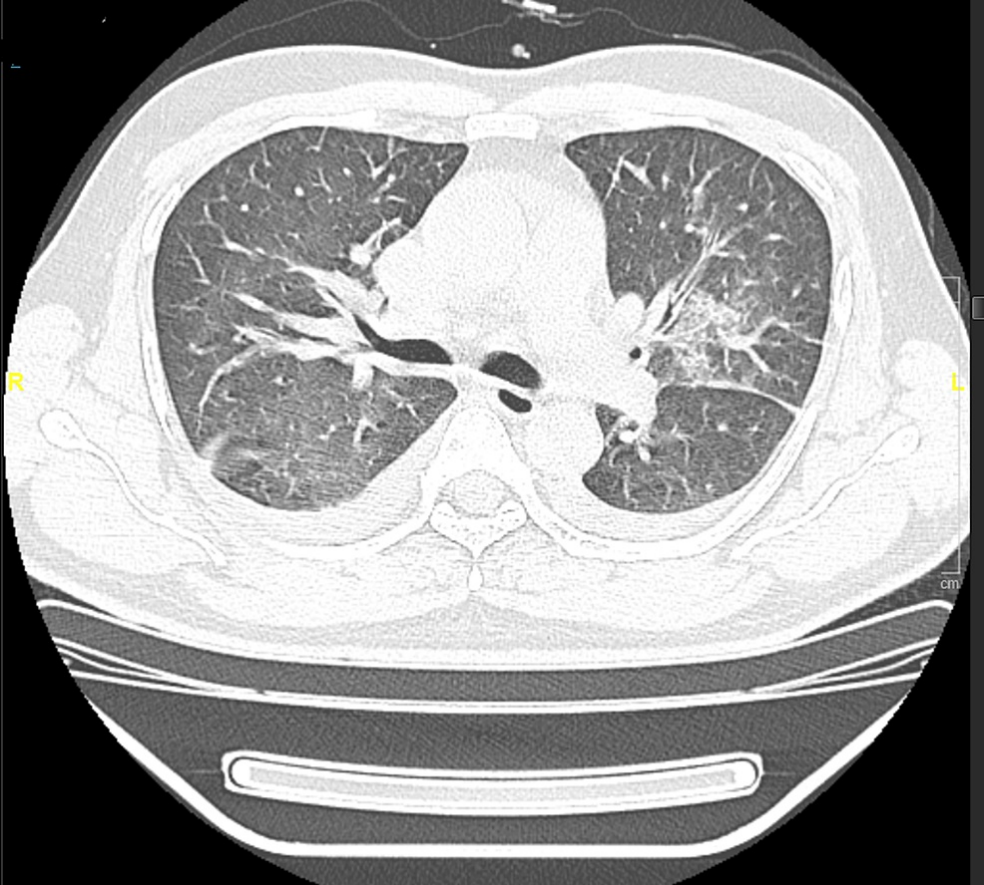
CT chest on the day of admission showing bilateral few small-sized patchy areas of consolidation and ground-glass opacity.

The limbs were warm with no remarkable skin lesions, no pedal edema, or calf tenderness. The remainder of the examination was normal. His urine routine and culture did not show any evidence of sepsis. HIV was negative. His investigations (Table 1) and Computed Tomography (CT) chest (Figure 1) on the day of admission are shown below.

**Table 1:** Initial Laboratory Investigations WBC: white blood cell; CRP: c-reactive protein; TSH: thyroid stimulating hormone

Investigation	Result	Reference Range
WBC	10.72 x10(3)/mcL	4.00-11.00 x10(3)/mcL
Neutrophil	74.7 %	40.00-80.00 %
CRP	28.5 mg/L	0.00-03.00 mg/L
Lymphocytes	14.70 %	18.00-42.00 %
Procalcitonin	0.16 ug/L	<= 0.10 ug/L
Anion gap	8.00 mmol/L	12.00-20.00 mmol/L
Sodium	140 mmol/L	135–145 mmol/L
Potassium	3.67 mmol/L	3.6–5.1 mmol/L
Blood glucose	8.3 mmol/L	3.9-10 mmol/L
Blood urea	7.8mmol/ L	4-8 mmol/L
Osmolality	226 mosm/L	275-285 mosm/L
Urine osmolality	348mosm/L	50-1200 mOsm/kg
Spot urine sodium	42 mmol/L	>20 mmol/L
TSH	3.4 mIU/L	0.55 -4.78 mIU/L
AM Cortisol	465 nmol/L	138-635 nmol/L

**Figure 2:**
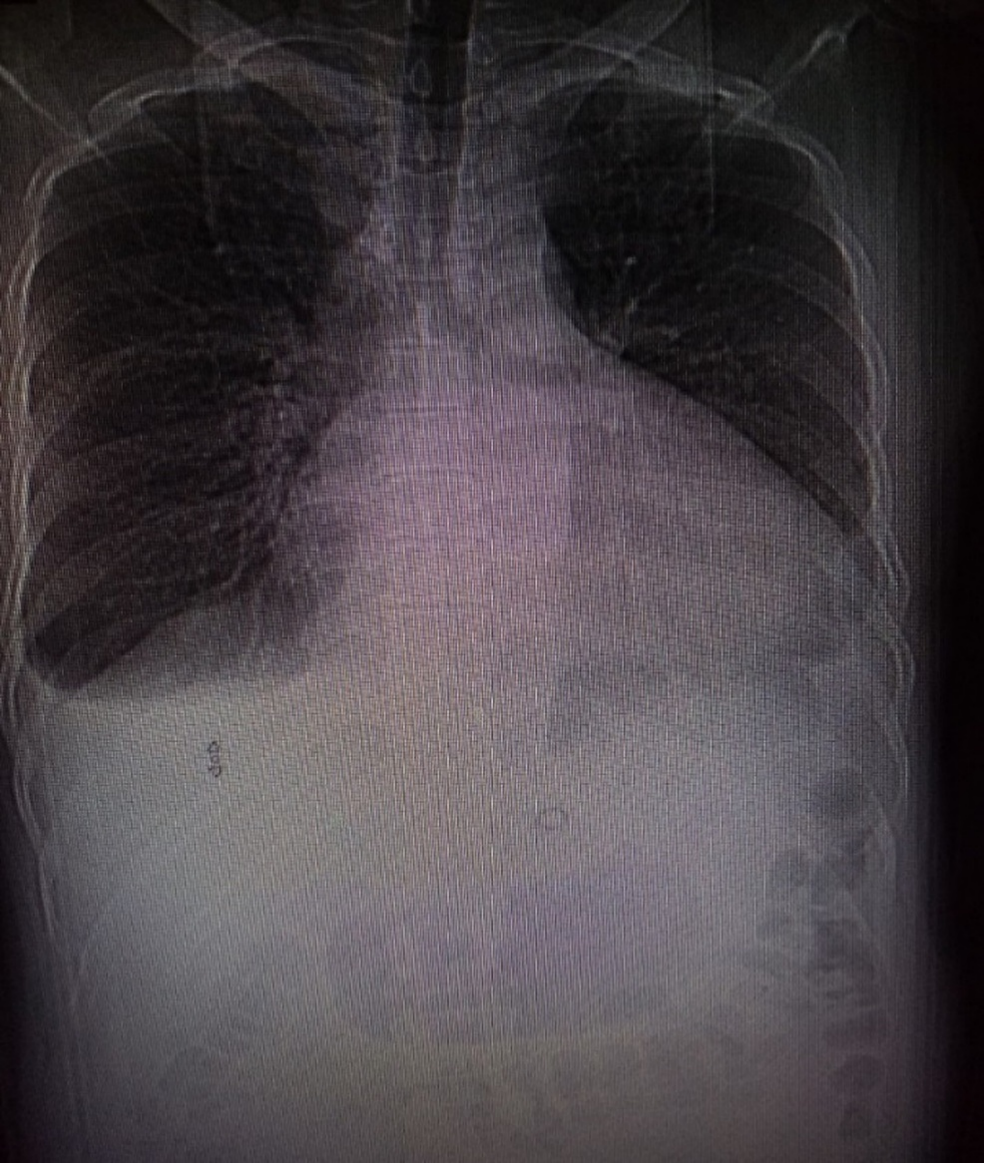
X-ray chest on day six of admission to ICU: showing minimal right-sided pleural effusion

**Figure 3:**
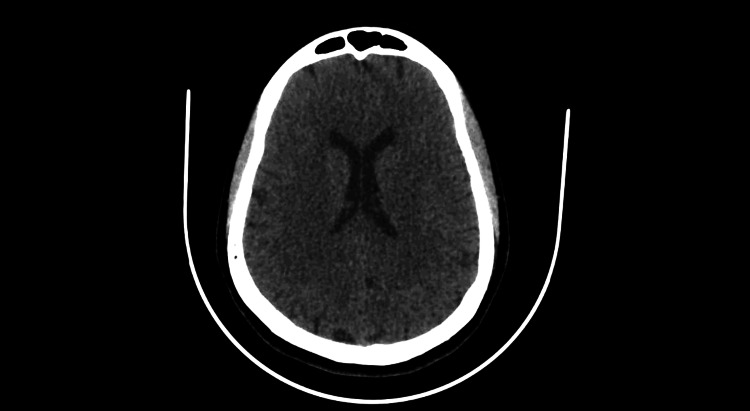
CT Brain showing no abnormal changes.

The patient was started on lopinavir/ritonavir and hydroxychloroquine tablets for the treatment of COVID-19 as per the local therapeutic protocol. The general condition of the patient was stable till the fifth day of admission, when he started to complain of epigastric pain, nausea, and vomiting. Lactated ringer infusion 500ml and pantoprazole injection IV 40 mg were given. There was no hypotonic solution included in his treatment. However, a few hours later, the patient started to feel dizzy; a blood sample was taken for a complete blood count and metabolic panel. He looked drowsy and disorientated and eventually had an episode of loss of consciousness. His vitals were within normal limits, and no focal neurological deficit. CT Brain revealed no abnormalities. On reviewing the result of his investigations, his sodium level was critically low, i.e., 105 mmol/L and glucose level 7 mmol/L, two days before his plasma sodium was 140mmol/L and glucose level 8.3 mmol/L. His chest x-ray showed minimal pleural effusion (Figure 2) and CT brain was normal (Figure 3).

The patient was treated in the ICU with hypertonic saline 100 ml twice, and 50 ml added later with continuous monitoring of his plasma sodium. This was followed by fluid restriction to 1000ml per 24 hours. After 24 hours in ICU, his sodium rose to 117 mmol/L and glucose level 7 mmol/L. He stayed in the ICU for three days, and then he was shifted to the medical ward with a serum sodium level of 134 mmol/L and a glucose level of 6.5 mmol/L with no residual neurological deficits.

Tazobactam/piperacillin and doxycycline were added empirically; the sepsis screen was negative later. The minimal pleural effusion was treated conservatively without the need for aspiration.

The patient’s symptoms of dizziness, disorientation, and cough resolved. He neither required mechanical ventilation nor inotropic supports as he maintained oxygen saturation target levels with a simple face mask, and he was hemodynamically stable. He tested negative for COVID-19 and was discharged home without any obvious residual complications.

## Discussion

Hyponatremia is the most common electrolyte disorder seen in daily clinical practice. It is estimated that around 15-30% of all hospitalized patients with electrolyte imbalance are hyponatremic [4]. Almost 50% of this hyponatremia occurs during a patient’s hospital stay [5]. Hyponatremia, which is defined as the plasma sodium concentration of less than 135 mmol/L, multiple factors that disrupt water and sodium homeostasis can lead to hyponatremia. The diagnosis and management of hyponatremia could be thought-provoking in the acutely ill patient. An organized approach comprising of a thorough history and physical examination is central to define the volume status. Obtaining plasma sodium and osmolality coupled with urine sodium and osmolality levels will narrow the list of causes of hyponatremia.

Patients with SIADH have a composite of two elements: ADH-induced water retention together with solute loss. Several pulmonary diseases, particularly pneumonia, as seen here in this patient, can lead to SIADH by unknown mechanisms. The diagnosis of SIADH in this patient was established as being euvolemic, hypotonic hyponatremia with normal thyroid and adrenal functions. Many studies have reported a relation between COVID-19 and SIADH-induced hyponatremia and associated neurologic manifestation [6,7].

In our patient other causes of hypotonic hyponatremia were ruled out as the patient had neither history of psychiatric disorder e.g. primary polydipsia, nor taking medication that might give a similar biochemical picture e.g. selective serotonin reuptake inhibitors. He had not been on diuretic therapy for his hypertension and denied using illicit drugs.

Interleukin-6 (IL-6) is linked to COVID-19 due to the inflammatory surge caused by the viral infection. An increase in IL-6 causes a drop in the sodium levels which in turn leads to non-osmotic ADH release and SIADH in COVID-19 patients [8]. Ischemic and hemorrhagic stroke in patients with COVID-19 has been reported in the past. However, in our patient the CT scan of the head was normal ruling out coagulopathy to be a potential cause, further confirming SIADH-induced encephalopathy [9]. The interval of hypertonic saline treatment of acute, life-threatening hyponatremia should be determined by the improvement in the patient’s symptoms and signs. Our patient showed drastic improvement in his symptoms after treatment with hypertonic saline and thus we decided to not repeat any brain imaging. Patients with acute hyponatremia are typically fatigued, drowsy, agitated, and complaining of anorexia, nausea, and/or vomiting [10]. Although blood urea has a limited increase effect on serum osmolality, it is notably sensitive to hypovolemia. Therefore, its level can be used in the workup of determining the type of hyponatremia [11]. As per the above discussion, we believe that the inflammatory response due to the ongoing COVID-19 infection and non-osmotic ADH release mediated by IL-6 contributed to hyponatremia in our patient further causing encephalopathy.

## Conclusions

SIADH-induced encephalopathy and COVID-19 infection is a very rarely presented manifestation although reported earlier. The key pathophysiology as discussed above is known to inflammatory mediator IL-6 affecting the sodium levels and causing electrolytes imbalance. It can be confused with a hypercoagulable condition caused by COVID-19 however, electrolyte imbalance and hyponatremia should always be in the differentials in a patient presenting with deranged mental status and COVID-19 infection.
